# Photobiomodulation Dose Parameters in Dentistry: A Systematic Review and Meta-Analysis

**DOI:** 10.3390/dj8040114

**Published:** 2020-10-06

**Authors:** Mark Cronshaw, Steven Parker, Eugenia Anagnostaki, Valina Mylona, Edward Lynch, Martin Grootveld

**Affiliations:** 1 Leicester School of Pharmacy, De Montfort University, Leicester LE1 9BH, UK; steven.parker@my365.dmu.ac.uk (S.P.); eugenia.anagnostakis@my365.dmu.ac.uk (E.A.); vasiliki.mylona@my365.dmu.ac.uk (V.M.); edward.lynch@hotmail.com (E.L.); mgrootveld@dmu.ac.uk (M.G.); 2 School of Dentistry, College of Medical and Dental Sciences, University of Birmingham, Birmingham B5 7EG, UK; 3 School of Dental Medicine, University of Nevada, Las Vegas, NV 89154, USA

**Keywords:** diode laser, low-level laser therapy (LLLT), dentistry, photobiomodulation (PBM), systematic review

## Abstract

**Objective**: This systematic review and meta-analysis of published randomized controlled trials examines a possible relationship between optical spot size at surface tissue, irradiance, radiant exposure, total energy delivered, operator technique and reported clinical outcomes. **Background**: Clinical photobiomodulation (PBM) therapy has achieved a high level of evidence-based acceptance in the mitigation of oral mucositis associated with cancer radiotherapy and chemotherapy, and supportive clinical research in relation to orthodontic tooth movement, oral medical conditions, including burning mouth syndrome, xerostomia and lichen planus. Inconsistent outcomes have been reported not withstanding a substantial body of primary supportive research from clinical, in vitro and animal studies. **Materials and Methods**: PubMed, Cochrane Database of Reviews and Google Scholar search engines were applied to identify human clinical trials of PBM therapy in clinical dentistry. A total of 766 articles between February 2009 and June 2020 were identified and following a full text evaluation, 38 papers with sufficient data to permit analyses are included in this investigation. **Results**: Following a detailed assessment of potential factors that may have an influence in clinical outcome, a clear trend is apparent associating optical spot size to a positive or negative effect. Furthermore, there is a clear difference in the reported results in relation to total energy applied, delivery techniques and optical parameters, which merits further investigation. Factorial statistical analyses identified an association between smaller optical surface applications and an overall lower level of reported clinical success in treating superficial and deeper targets, and correspondingly sub-surface larger target tissues were found to be more responsive to therapy by use of a larger optical surface spot size. Moreover, use of multiple small diameter probe applications was found to provide inconsistent results. **Conclusions**: Many factors can confound clinical success including variations in anatomy, site location, clinical condition and subject individuality. To achieve higher levels of predictable outcome, a mature appreciation of these factors, plus an expanded understanding of laser parametry, tissue volume and target depth to deliver an adequate dose within current recommended guidelines, is essential.

## 1. Introduction

Following over fifty years of continued research, a considerable body of evidence has accumulated in relation to the clinical effects of light and particularly laser light on biological tissues [1,2]. There are many reported successes in a variety of clinical oral conditions including pain control in orthodontics, the mitigation of aphthous ulceration, the management of dentinal hypersensitivity as well as the prevention and mitigation of cancer radio- and chemo-therapy-related oral mucositis [3,4,5,6,7,8,9,10]. Further efforts spurred on by the reported clinical successes in these diverse clinical uses have been attempted with some promising results in the potential acceleration of the rate of orthodontic tooth movement, as well as an aid to many oral medical conditions, including burning mouth syndrome, xerostomia and lichen planus [11,12,13]. Moreover, there has been considerable interest from the oral surgery community in relation to reducing post-operative pain, trismus and swelling following third molar extractions, as well as in relation to conditioning tissues to achieve optimal healing and regeneration of tissues [14,15,16,17]. Photobiomodulation (PBM) therapy would appear to offer many possible clinical benefits [18,19,20,21,22].

The mechanisms associated with PBM may be considered as operating at local, regional and systemic levels. Intracellular responses include an increase in activity in mitochondrial metabolism resulting in the elevated production of adenosine triphosphate (ATP), nitric oxide (NO) and reactive oxygen species (ROS). It has been theorized that a photo-induced ROS stress response may be an associated element in laser-induced analgesia [1,23,24].

The cellular targets for PBM include the inhibition of cyclooxygenase resulting in the reduced production of prostaglandins, which act as key mediators of the acute inflammatory response. Moreover, other highly significant anti-inflammatory pathways have been characterized [25,26]. Consequent to the recognized analgesic and anti-inflammatory actions of PBM, it has been proposed that PBM offers a tool that may permit a locally directed alternative to systemic drugs without the attendant risks of allergy, toxicity, impaired healing and other important medical issues such as addiction [27,28]. There is a dose-related response which in our view is best described as a multiphasic outcome, as at relatively low doses of radiant exposure there can be photobiostimulation and at higher levels photobioinhibition. The former is associated with enhanced healing, whereas the latter has been found to be optimal for pain relief [29,30,31,32,33,34].

PBM is in essence a non-surgical therapy associated without any significant tissue hyperthermia. When the dose is delivered with a small optical spot size probe, this requires a low power output to avoid inadvertent tissue damage. Diode lasers used in dentistry use a TEM_00_ mode as standard with a fiber optic delivery system. In consequence, there is a beam divergence at the fiber end typically of approximately 15 degrees in sum total. By adjusting the distance of the optic probe from the target, using the visible wavelength aiming beam as a guide, it is possible to create a surface spot size with or without a non-initiated surgical tip in place approximating up to 1 cm or more in diameter [35].

The spatial beam profile is inherently Gaussian, which is of minimal significance with a very small spot size. As the diameter of the spot increases, then as the energy profile in the mid third of the beam is 2–4 times that of the periphery, this becomes an important factor to take into account whilst attempting to deliver a predictable unit dose to the target tissues. To overcome this issue, there are dedicated PBM handpieces which can optically correct the beam profile to a flat top shape with collimation. Alternatively, in an attempt to more evenly distribute the delivered energy, a scanning technique may be adopted [2,36].

In an earlier analytical review, we identified many issues related to the reportage of parameters in the published evidence base [37]. This paper seeks some clarity amongst the very many published studies to identify factors related to dose and dose delivery that may be significant to reported outcomes. At present, there is no consensus on wavelength or delivery methodology. We believe this review is the first to address the question of whether or not surface spot size, operator technique and volumetric delivered energy may be of significance in clinical outcomes.

## 2. Materials and Methods

The search engines PubMed, Cochrane and Google Scholar were used with following keywords and combinations:

Laser AND (Photobiomodulation OR PBM OR LLLT OR Low level) AND (soft tissue OR oral surgery OR lip OR tongue OR buccal mucosa OR lichen OR TMJ OR oral mucositis OR orthodontics).

After applying the additional filters (Clinical Trial [ptyp] AND “last 10 years” [PDat] AND Humans [Mesh] AND English [lang]), the initial number of 6315 articles was reduced to 246.

We also screened the references of recent systematic reviews to identify additional, original studies that were not retrieved in our prior search. In accordance with the PRISMA statement, case reports and non-randomized controlled trials were excluded [38].

### 2.1. Search Strategy

The eligibility criteria applied followed the qualitative analysis tool PICOS [38]:

- Population = Patients receiving PBM therapy as adjunct to treat presenting pathology.
- Intervention = Administered PBM to assist in pain management/healing.
- Compared with = Control groups receiving alternative therapy/sham phototherapy.
- Outcome of interest = Pain; Healing; etc.
- Study type = Randomized Controlled Trials.

Two reviewers independently screened titles and abstracts (MC and SP). In the event of disagreements, this was resolved by discussion. Inclusion/exclusion criteria were applied as follows:

Inclusion criteria:

- Randomized controlled clinical trials;
- Laser applied as an adjunctive therapy;
- Standard orthodox treatment performed to all groups;
- Negative control group;
- Minimum of 10 participants per group.

Exclusion criteria:

- PBM therapy not applied;
- Duplicates or studies with the same ethical approval number;
- Alternative to control conventional treatment applied to the test group;
- Less than 10 subjects per group;
- Clinical trials, case series, pilot studies, (not randomized controlled);
- In vitro studies;
- LED rather than laser as light source.

Subsequent to the screening and implementation of the eligibility criteria, 38 articles were included, which were segregated in terms of:

Optical spot sizes in the range of 0.02–0.08 cm^2^ (group A–16 articles)
Optical spot sizes in the range of 0.126–0.38 cm^2^ (group B–9 articles)
Optical spot sizes in the range of 0.51–4 cm^2^ (group C–13 articles).

The search was performed from 8 April to 15 June 2020.

**Figure 1:**
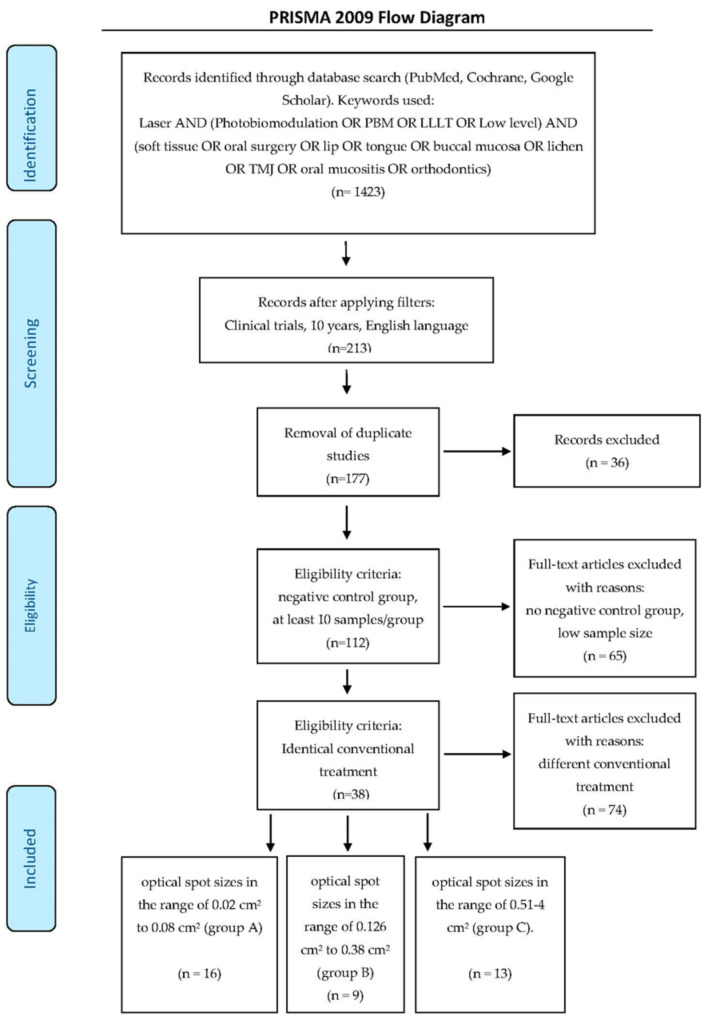
PRISMA flow-chart of selected criteria for the included study reports. [39].

The details of the PRISMA selection criteria are presented in Figure 1.

### 2.2. Quality Assessment

Furthermore, studies were subject to a risk of bias assessment (Table 1). The Cochrane Risk of Bias tool [40] was modified and applied according to the requirements of this systematic review.

A determination of the risk of bias was made to each study according to the number of “yes” or “no” answers to the following questions:

- Randomization?
- Sample size calculation and required sample number included?
- Allocation ratio of 1:1?
- Baseline situation similar?
- Blinding (single/double)?
- Parameters of laser use correctly described, and calculations checked?
- Power meter used for calibration of the source?
- Numerical results available (statistics)?
- Outcome data complete?
- Correct interpretation of data?

According to the total number of “yes” answers to the above questions, a classification was performed. The degree of bias was calculated as follows:

- High risk: 0–4.
- Moderate risk: 5–7.
- Low risk: 8–10.

Three of the authors analyzed the included articles and a consensus was arrived at as to the degree of scientific rigor of the published data. As a result of this assay, all of the papers were considered medium to low risk in terms, scoring between 6 and 10 against the series of selected criteria. Given the heterogenous nature of the included studies, for the statistical analysis to have meaning we measured any variation from baseline between the control groups as well as the test groups. A score on a scale of 0–5 was employed. This scale was employed as a prequel to the reported net outcome in the test groups. A grade of zero = zero change, 1 = up to 20% change to baseline, 2 = up to 40% change to baseline, 3 = up to 60% change to baseline, 4 = up to 80% change to baseline and 5 = 80% or more. The difference between the control group variation and the test group variation is the figure reported in the tables as the net outcome.

Reported net outcomes were graded on a score of zero to five, where zero reflects a statistically insignificant outcome, one a statistically significant low result (<20%), two a medium level of significant response (20–40%) and three a higher level of difference (40–60%) and four a highly significant difference = 60 − 80%, and five ≥80%. Many studies employed a visual analogue scale to record patient perceptions of pain and here a score of difference of 1–2 points is treated as level one, 2–4 points as level two and 5 or 6 as level three, 7 or 8 as level 4 and 9 or 10 as level 5. Calculations were made of total energy delivered, fluence and surface area exposed to the beam. Studies employing a multiple point method as well as those that used a scanning technique were identified. Sub-surface target tissues of 5 mm or more are listed and evaluated in respect to the total dose delivered and surface area exposed to the therapeutic dose. Data were extracted from the selected papers on target depth, target size, total energy delivered, radiant exposure (J/cm^2^), and the surface tissue area exposed to the tip. 

## 3. Statistical Analysis of Systematic Review Clinical Outcome Dataset

For the complete dataset acquired, which was based on the systematic review of a total of 38 published reports, an analysis of variance (ANOVA)-based experimental design was employed for its statistical analysis. For this purpose, both spot size (small, medium or large) and target tissue depth (classified as superficial or deep) were featured as fixed effects at 3 and 2 classes of classification, respectively, and the model was therefore a 2-factor system (Equation (1)). In this equation, y*_ijk_* represents the clinical score outcome response (dependent) variable (i.e. difference observed between the mean laser treatment and control group clinical outputs), µ the null hypothesis population mean value in the absence of all sources of variation, and S*_i_*, T*_j_*, and ST*_ij_* represent the ‘between-spot sizes’, ‘between-target depth’, and the spot size × target depth interaction effects, respectively; e*_ijk_* represents the unexplained error source of variation. The above second-order interaction effect was incorporated into this experimental design in order to explore any differential responses of superficial and deep tissue target sites to the three treatment spot sizes applied.

y*_ijk_* = µ + S*_i_* + T*_j_* + ST*_ij_* + e*_ijk_* (1)

Primarily, the mean control group outcome scores were subtracted from those of the laser treatment groups for each study, and then the nominal constant of 1.00 was added to all such difference values. Prior to analysis, these (1 + Δ) values (where Δ = mean clinical outcome score differences between the treatment and control groups for each study incorporated) were weighted with a standardized derivative of the total number of participants involved in each reported study (n = 16 − 239, mean ± SD 58.4 ± 43.0). This weighted (1 + Δ) dataset was then log_10_-transformed in order to satisfy assumptions of variance homogeneity and normality, and the above ANOVA model was applied to determine the statistical significance of any differences or effects found. *Post-hoc* analysis of differences between individual factor classification mean values for this model was performed using a Bonferroni testing system.

Prior tests for intra-group sample variance homogeneity (Bartlett’s and Levene’s tests) and normality (Jarque–Bera test) assumptions for each classification (log_10_ (1 + Δ) response variable dataset) showed no significant deviations from these.

## 4. Results

In total, 38 studies were included with sufficient data to permit statistical analyses. Details of the data extraction are shown in Table 2, Table 3 and Table 4. Sixteen employed optical spot sizes in the range of 0.02–0.08 cm^2^ (group A) [40,41,42,43,44,45,46,47,48,49,50,51,52,53,54,55] with a further nine applying surface optical spot sizes in the range of 0.126–0.38 cm^2^ (group B) [56,57,58,59,60,61,62,63,64]. Thirteen studies employed optical spot sizes in the range of 0.5–4 cm^2^ (group C) [65,66,67,68,69,70,71,72,73,74,75,76,77].

In the studies employing a small optical spot, delivered total energy to a single point ranged from 0.12 to 18 J, whereas the total energy delivered for this same sub-set of studies ranged up to 244 J. In comparison, group B studies using a medium optical spot size, administered a dose of 0.3 to 18 J at a single point with a total delivered energy up to 116 J. In the group C studies using large optical spot sizes, the total delivered dose at a single point ranged from 1.8 to 300 J. 

**Figure 2:**
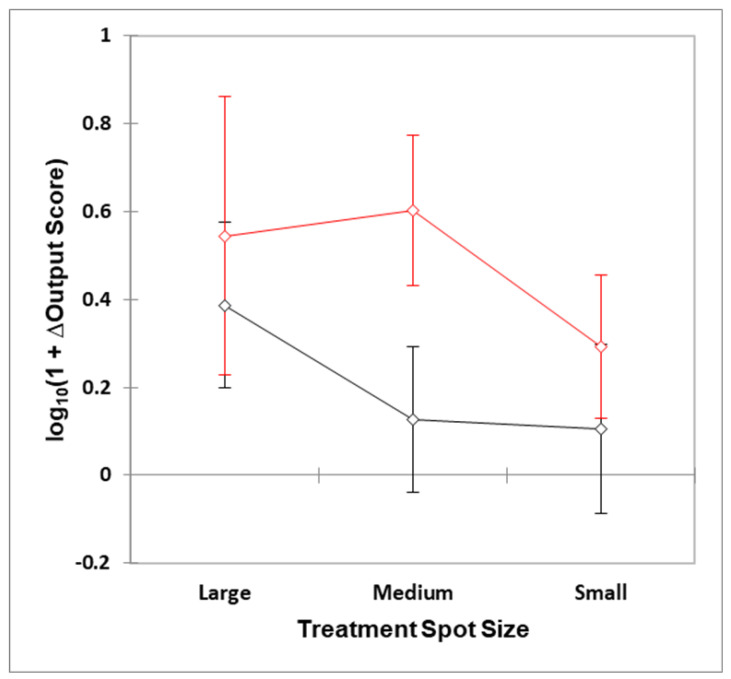
Plots of mean ±95% confidence intervals for the large, medium and small spot size treaments at both deep (black) and superficial (red) target sites. ΔOutput Score represents differences determined between the mean clinical outcome score variables observed for the laser and control treatment groups, which was positive for all 38 studies reported.

The statistical analyses identified a correlation between smaller optical surface applications and an overall lower level of reported clinical success in treating superficial and deeper targets (Figure 2). In addition, sub-surface larger target tissues such as salivary glands, muscles of mastication and the temporomandibular joint were found to be more responsive to therapy by use of a larger optical surface spot size. The use of multiple small diameter probe applications was found to be associated with inconsistent results. The optimum outcome for smaller spot size studies was associated with the oral mucositis studies which employed multiple points of application and a corresponding higher overall dose to the target area.

ANOVA demostrated that both the ‘between-spot sizes’ and ‘between-target depth’ factors were both highly significant effects (*p* = 8.45 and 5.72 × 10 − 3 respectively), whilst the second-order spot size × target depth interaction source of variation was not so (*p* = 0.33). The nature of these differences are visible in Figure 2, which shows mean ±95% confidence intervals for the three spot size treatment options applied at deep or superficial target tissue sites. *Post-hoc* contrast analysis performed via the Bonferroni approach revealed that there were significant differences between small vs. large spot sizes both applied at the deep target site, (*p* = 0.031), i.e., large > small; small vs. large spot sizes both applied at the superficial target site (*p* = 0.035), i.e., large > small; small spot size/deep target site vs. medium spot size/superficial target site (*p* = 0.0084); small spot size/deep target site vs. large spot size/superficial target site (*p* = 0.0072); medium spot size/deep target site vs. medium spot size/superficial target site (*p* = 0.0013); and medium spot size/deep target site vs. large spot size/superficial target site (*p* = 0.0021).

Differences observed between the mean log_10_(1 + Δ) large and medium spot size values were not found to be statistically significant for both deep and superficial target sites, although the former comparison was very close to a *p* value of 0.05 (*p* = 0.056).

## 5. Discussion

Our findings show a clear association between the application of a larger surface optical spot size (groups B and C) and an optimal clinical outcome for deeper targets. Indeed, our analysis showed that, for superficial targets, although all three groups may be associated with a successful outcome, there is an evident significant difference between the groups A, B and C. The usage of a small spot size (group A) was found to be ineffective in the management of deeper pathologies by comparison to larger spot sizes (groups B and C). 

In dentistry, target tissues for PBM generally rest in the range of surface to around 1 cm in depth. However, attaining a meaningful dose to reach the target at a cellular level at sub-surface depth can pose a challenge in the absence of evidence-based guidance to the clinician in the choice of the best parameters and technique. A consistent high level of success has been recognized in the management of oral mucositis associated with cancer chemotherapy and radiotherapy. It is perhaps no coincidence that a condition associated with superficial ulceration has been the first to achieve the highest grade of level of evidence acceptance [10].

Evidence based optimal dose bands have been proposed to achieve beneficial clinical effects in PBM therapy. Notwithstanding many carefully designed studies and the determined efforts of dedicated researchers, achieving consistent high level outcomes in many clinical dental conditions has proven elusive.

**Figure 3:**
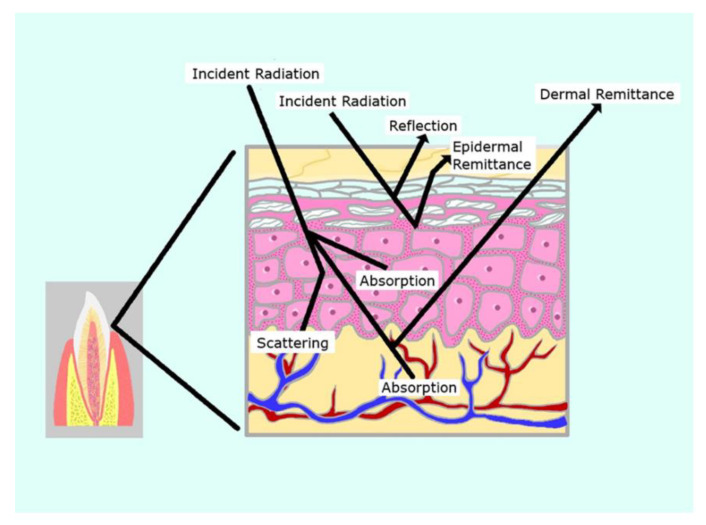
Outcomes of optical transport at a target tissue result in significant energy loss (radiant exitance).

The required dose is in essence a function of power output, optical footprint within the tissues and application time along with a consideration of the volume and depth of the tissue target. On striking the surface, 4–7% of the applied photonic energy is reflected depending on the angle of incidence. In addition, due to remission and internal refraction and back transmission, there is a level of radiant exitance (energy loss) that amounts to an appreciable portion of the surface application (Figure 3). A recent in vivo study by Alvarenga et al. of gingival transillumination using a 660 nm laser found that this amounted to around 50% power loss at 5 mm in depth [79]. Tissue consistency and optical transmission vary according to tissue type as well as the presence or absence of oedema, erythema and the thickness of the epidermis or the presence of any dark pigmentation. In consequence, power losses by radiant exitance between subjects can differ, which may be significant in dental PBM therapy, particularly when an extra-oral approach is adopted.

The typical wavelengths used in PBM fall within the red to near infra-red (NIR) range of 650–1200 nm. Due to the relatively poor absorption of these wavelengths in biological tissues, there can be significant optical transport within the tissues. This is, however, subject to a high degree of photon scattering due to the coincidence of these wavelengths with the size of tissue components at a sub cellular level [80,81]. As a result, there can be a significant degree of forward, back and, to a lesser degree, lateral scatter, which attenuates the penetration to depth. However, it is anticipated that at a depth of 1 cm there will remain around 5–10% of the surface NIR photons arriving at a possible target at this level [82,83]. In view of the energy loss as described above, it is recommended that there should be an increase in the surface applied dose to around 10 times for sub-surface targets. For example, to deliver a dose to target which is 1 cm in depth of 5 joules/cm^2^, a surface application of 50 J/cm^2^ is suggested [84]. 

To assist future efforts in this regard, it is of paramount importance that details be provided of the tip diameter and the operator mode of use, contact or at a measured distance, static or scanning. To assist reproducibility of studies, the use of a power meter to calibrate the exposure is important. Moreover, information regarding the beam divergence angle, the beam profile (flat top or Gaussian), plus the number of points treated as well as the total time for the procedure is required to be reported. Finally, a declaration of the treatment site depth location and the approximate target tissue size would also be appropriate [37].

Many of the studies included in this review applied individual spot applications with a fluence of 0.24 to 225 J/cm^2^. Given multiple points of application, this would at first sight appear to address the twin need for a higher dose to reach depth as well as to cover larger volumes of sub-surface tissue targets. Many studies utilized small optical spot sizes that had been applied to treat both small superficial as well as larger deeper tissue targets. As identified in Table 2 studies adopting this approach have been calculated here as delivering to target a sum of 0.6 to 225 Joules. The size of tip areas employed in the clinical trials in Table 2 range from 0.02 to 0.08 cm^2^. In contact with the tissues at very low levels of irradiance, this can be calculated to achieve levels of radiant exposure which may appear consistent with recommended dose guidelines of 2–10 J/cm^2^ for optimal cellular productivity or 10–30 J/cm^2^ for analgesia. Where multiple points of application were used, the total surface area exposed to the small diameter probe ranged from 0.04 to 3.2 cm^2^. Our analysis suggests that a limited area of exposure and the delivery of a low overall sum of energy can result in a compromised clinical outcome, particularly in deeper tissue targets.

As part of our current research, measurements of optical spatial power distribution are being taken of series of varied in depth measured millimeter thicknesses of porcine lean muscle tissue samples. This involves the application of a calibrated laser source trans-illuminating the target tissue and the emergent beam is measured using an Ophir beam profilometer. Software analysis using BeamGage Standard 5.5 provides data and visual images of a 2D and 3D render of the optical irradiance in a color rainbow range of mW/cm^2^ red (high) through to violet (low).

**Figure 4:**
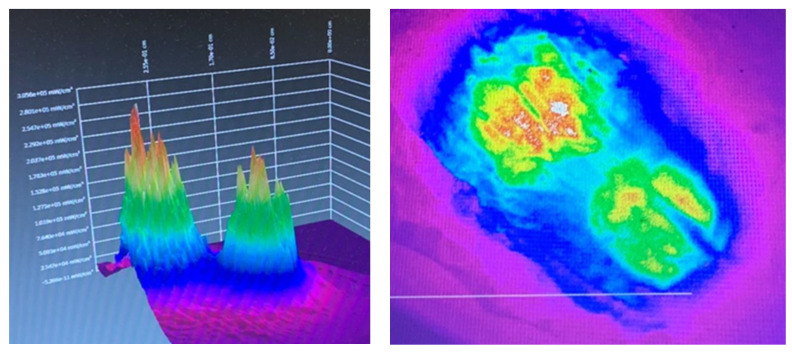
Three-dimensional render (**left**) and planar view (**right**) of the beam profile of two parallel optic beams 1 cm apart applied to a tissue sample. These images are offered as a guide to the reader to demonstrate the lack of lateral spread from the tips. Note the Gaussian distribution and the relative lack of lateral scatter.

Figure 4 shows a planar and three-dimensional rendering of the optical transmission and spatial beam profile of two parallel 2 mm tipless 940 nm surgical handpieces placed 1 cm apart in near contact to a porcine tissue sample of 2 mm in thickness at an output power of 500 mW. The resulting images show two distinct peaks of energy with very little lateral scattering. This implies that a multiple point technique will only deliver energy to a small volume of tissue and furthermore, due to attenuation, the area treated at depth is likely to be small.

If a small optical spot size is used, then the overall dosimetry may fall far short of the intended dose to the target level. The outcome of this method has been identified here as being inconsistent, and our statistical analysis demonstrates that this approach is associated with a higher incidence of either a null or a lower level effect in treating sub-surface conditions. 

A further problem highlighted by our study relates to eliciting a successful response in large sub-surface tissue targets. For example, the muscles of mastication associated with myalgia include some relatively large areas and volumes of tissue. Similarly, the salivary glands targeted for PBM therapy to stimulate salivary flow are areas of tissues that occupy a significant volume. It is our contention that, based on the positive outcomes identified here, the use of a larger surface optical foot print as opposed to a small optical spot size, along with a corresponding increase in the overall level of delivered energy to compensate for optical attenuation, may offer the prospect of increased clinical success. 

In respect of the dose, this is usually described as fluence in Joules/cm^2^; however, the challenge is to deliver the essential cellular level of photonic input to depth and volume in Joules/cm^3^. This may be the source of error in dosimetry, which may have arisen as a consequence to a high degree of confusion in the literature, as identified in respect of nomenclature by Hadis et al. [85]. Based on our findings here, we propose that a failure to take into account tissue volume and depth may lead to a disappointing clinical outcome. Moreover, to cover large areas of sub-surface tissues, either a scanning technique or multiple overlapping spots may be used. Or, as an alternative, a large surface optical spot can be used. This latter approach saves time as a larger area can be treated more quickly. Moreover, with a larger optical spot size, the overall energy delivered to the target is greater, whilst still keeping the therapeutic dose to the recommended range of 2–8 joules/cm^2^ for enhanced healing and 10–30 joules/cm^2^ for analgesia. The volume of tissues treated is increased and we propose that this may be associated with an improved clinical outcome.

In a study of healing and pain associated with connective tissue grafting, Dias et al. achieved a good result using a 0.03 cm^2^ spot size at a power output of 30 mW delivering 0.12 J/point with a total of 5 points of application to the graft [46]. Neves et al., in a similar study, applied a 0.03 cm^2^ spot size at the same power output of 30 mW delivering 0.45 J/point and 0.9 J/point to a total of two points. However, Neves reported a modest tissue response at 7 days in the higher dose group and statistically no significant differences at all other times between the treatment and sham groups [47]. In the Dias study, the total area treated was 5 × 0.03 cm^2^, which is 0.15 cm^2^, with a radiant exposure of 0.6/0.03 = 4 J/cm^2^. By contrast, Neves treated 2 × 0.03 cm^2^, which is 0.06 cm^2^ with a radiant exposure of 0.9/0.06 = 15 J/cm^2^ with a further group at higher energy settings of 1.8/0.06 = 30 J/cm^2^. The difference in result between these studies may be due to the area treated, as two points of application may be insufficient to cover the target and/or possibly the radiant exposure employed by Neves et al. (15–30 J/cm^2^) was higher than the optimum for healing of 2–8 J/cm^2^. 

We surmise that the use of a small optic probe may be beneficial to treat a small aphthous or herpetic ulcer providing the wound area is covered and an adequate dose applied. Indeed, this method has enjoyed considerable success in the prophylaxis of oral mucositis associated with chemotherapy and head and neck radiotherapy [10]. However, the management of deeper tissue targets of greater area may be compromised for the reasons described above.

**Figure 5:**
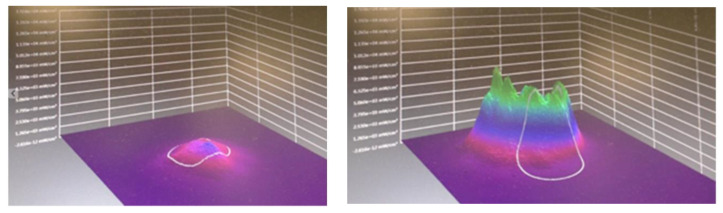
Three-dimensional render of optical beam profile: transillumination through a 2 mm sample of porcine tissue. **On the left**: 810 nm 0.28 cm^2^ spot size, 190 mW (0.5 J/cm^2^). **On the right**: 810 nm 1 cm^2^ spot size, 500 mW (0.5 J/cm^2^).

Figure 5 shows beam profilometer measures of optical energy distribution through a 2 mm thickness sample of porcine tissue. In the example shown, in 10 seconds at an output power of 190 mW, the target will have received 10 × 190 mW = 1.9 J, whereas, at 500 mW, in 10 seconds the delivered energy = 10 × 500 mW = 5 J. Although the radiant exposure is identical (0.5 J/cm^2^), it is apparent that both the volume and depth of optical transmission may be influenced by the choice of optical spot size. Aside from a difference in optical footprint, the overall delivered energy is markedly different. By use of a larger spot size, the average radiant exposure over an increased area can be maintained low, although it is possible to elevate the overall power output of the device. In consequence, more energy can be delivered to a larger area, which saves time as well as exposing an increased volume of tissue to a therapeutic non-toxic dose.

**Figure 6:**
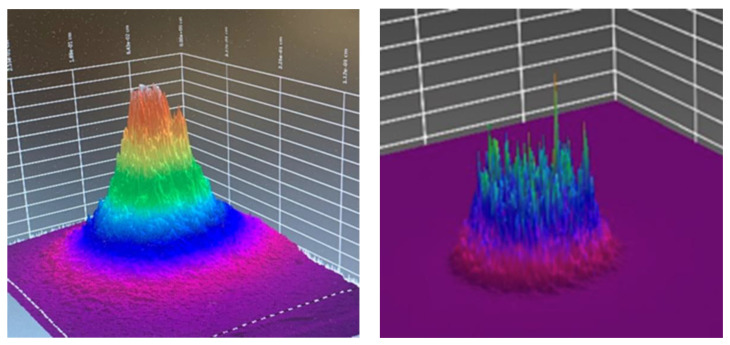
Gaussian beam energy profile (**left**) compared to an optically rectified flat top beam (**right**). Images are intended to display solely differences in optical spatial profile only (different optical sources and parameters).

The qualification to this principle is that the adoption of a larger surface spot size for dose delivery is not without its own potential attendant difficulties. Inherently, the emitted beam profile from a diode laser fiber optic cable is of a Gaussian distribution with an energy peak in the mid third of the beam, which is typically 2–4 times that of the periphery (Figure 6). A large applicator with a 3 cm diameter has a surface area of approximately 7.1 cm^2^. Set at a power output of 3.5 W, this would achieve an average irradiance of 500 mW/cm^2^. However, in the mid third of the beam, the radiant exposure and corresponding irradiance is 2–4 times the periphery, and this may produce a significant thermocline with the attendant risk of photo-induced hyperthermia. Khan and Arany investigated dose- and temperature-related laser-induced cellular stress and, based on their analysis, we regard it as important to avoid elevating tissue temperatures in excess of 43 °C to prevent potential tissue damage [86]. To overcome this difficulty, it is recommended that, with a large optical spot, a scanning technique be considered to avoid inadvertent superficial tissue overheating.

One alternative is the use of an optically corrected large spot size handpiece, which converts the top hat beam profile to a flat top (Figure 6) [87]. There is a relatively recent trend towards the use of so-called high-intensity laser therapy devices. To date, these have largely been applied in physiotherapy as well as in novel applications in respect of neurology; however, in the context of the current discussion here, it is noteworthy. A recent review by Salehpour et al. of transcranial PBM used to improve cognitive performance showed a consistent good outcome in eight out of nine studies [88]. All studies used large optical footprints of 1.4 to 22.48 cm^2^ with an overall higher energy delivery to the tissues, albeit at a level consistent with previous studies employing the same radiant exposure using a smaller spot size. 

Based on our analysis here, to deliver a meaningful dose at depth, it is suggested to apply a larger spot size. It is our belief that dose delivery requires a mature appreciation of optical physics in combination with an understanding of the treatment objective [32,37,80]. A limitation of our study is the lack of studies with adequate parameter reportage which reduces the power of our investigation. Moreover, at present, there are relatively few good-quality studies that have adopted our suggested methodology; although, we recommend this as an approach that is worthy of continued study. The use of PBM therapy as a science- and evidenced-based approach offers potential clinical gain to the discerning dental professional to compliment the recognized surgical benefits in dentistry offered by laser implementation [89]. Given the complexity represented by anisotropic tissues of variable depths and characteristics, it is challenging to supply a relatively straightforward clinical algorithm for deep tissue work. Consequent to the opportunity to analyze an extensive evidence base, it is our belief that this is now a realistic target to achieve

## 6. Conclusions

The review presented here highlights the difficulties to reconcile the recommended tissue level dosimetry to the condition. The outcome of this study supports the use of larger surface optical spot size applications for superficial and sub-surface treatment. In order to compensate for tissue attenuation at depth consequent to scatter and the resultant photon diffusion, it is recommended that higher surface dosimetry be adopted so that a realistic dose to the tissue target be delivered. An increase in surface area coverage can save significant time in the treatment of larger areas. Moreover, as the average radiant exposure over an increased area can be low, it is possible to elevate the overall power output of the device. In consequence, more energy can be delivered to a larger area, which both saves time and exposes an increased volume of tissue to a therapeutic non-toxic dose.

Finally, the finesse required in optimizing clinical outcome requires a proper appreciation of many factors, including optical transport, local tissue considerations as well as an understanding of the underlying mechanisms of PBM as a therapy. It is our view that a mature appreciation of oral anatomy and pathology, plus a well-developed understanding of laser/light transmission into biological tissues, is an essential requirement to a design for clinical success.

## Figures and Tables

**Table 1:** Risk of Bias.

**1. Risk of Bias for Small Spot Size Articles**											
**Citation [ref]**	**Random-Ization**	**Sample size Calculation and Required Number Included**	**Baseline Situation Similar**	**Blinding**	**Parameters of Laser Use Described and Calculations Correct**	**Power-Meter Used**	**Numerical Results Available (Stats)**	**No Missing Out-Come Data**	**All Samples/Patients Completed the Follow-Up**	**Correct Inter-Pretation of Data**	**Total Score/10**
**SMALL SPOT SIZE**											
Sugaya [41]	Yes	Yes	Yes	Yes	Yes	No	Yes	Yes	No	Yes	8
Valenzuela [42]	Yes	Yes	Yes	Yes	Yes	Yes	Yes	Yes	Yes	Yes	10
Barbosa [43]	Yes	Yes	Yes	Yes	No	No	Yes	Yes	No	Yes	7
Dias [44]	Yes	Yes	Yes	Yes	Yes	Yes	Yes	Yes	No	Yes	9
Neves [45]	Yes	Yes	Yes	Yes	Yes	Yes	Yes	Yes	No	Yes	9
Rezade [46]	Yes	Yes	Yes	Yes	Yes	Yes	Yes	Yes	Yes	Yes	10
Tuk [47]	Yes	Yes	Yes	Yes	Yes	No	Yes	Yes	No	Yes	8
De Carli [48]	Yes	Yes	Yes	Yes	Yes	Yes	Yes	Yes	No	Yes	9
Machado [49]	Yes	Yes	Yes	Yes	No	No	Yes	Yes	No	Yes	7
Magri [50]	Yes	Yes	Yes	Yes	Yes	No	Yes	Yes	Yes	Yes	9
Ramalho [51]	Yes	Yes	Yes	Yes	Yes	No	Yes	Yes	Yes	Yes	9
Oton-Leite [52]	Yes	Yes	Yes	Yes	Yes	No	Yes	Yes	No	Yes	8
Ferrante [53]	Yes	Yes	Yes	Yes	No	No	Yes	Yes	Yes	Yes	8
Nobrega [54]	Yes	Yes	Yes	Yes	Yes	Yes	Yes	Yes	Yes	Yes	10
Marin Conde [55]	Yes	Yes	Yes	Yes	Yes	No	Yes	Yes	No	Yes	8
Silva [56]	Yes	Yes	Yes	Yes	No	No	Yes	Yes	Yes	Yes	8
**2. Risk of Bias for Medium Spot Size Articles**											
**Citation [ref]**	**Random-Ization**	**Sample size Calculation and Required Number Included**	**Baseline Situation Similar**	**Blinding**	**Parameters of Laser Use Described and Calculations Correct**	**Power-Meter Used**	**Numerical Results Available (Stats)**	**No Missing Out-Come Data**	**All Samples/Patients Completed the Follow-Up**	**Correct Inter-Pretation of Data**	**Total Score/10**
**MEDIUM SPOT SIZE**											
Arduino [57]	Yes	Yes	Yes	Yes	No	Yes	Yes	Yes	No	Yes	8
Elbay [58]	Yes	Yes	Yes	Yes	Yes	Yes	Yes	Yes	No	Yes	9
Ramirez [59]	Yes	No	Yes	Yes	No	No	Yes	Yes	Yes	Yes	7
Landucci [60]	Yes	No	Yes	Yes	No	No	Yes	Yes	No	Yes	6
Moosavi [61]	Yes	Yes	Yes	Yes	Yes	No	Yes	Yes	Yes	Yes	9
Amanat [62]	Yes	Yes	Yes	Yes	Yes	No	Yes	Yes	No	Yes	8
Shirani [63]	Yes	Yes	Yes	Yes	Yes	Yes	Yes	Yes	Yes	Yes	10
Ang Khaw [64]	Yes	Yes	Yes	Yes	Yes	No	Yes	Yes	Yes	Yes	9
Antunes [65]	Yes	Yes	Yes	Yes	No	No	Yes	Yes	No	Yes	7
**3. Risk of Bias for Large Spot Size Articles**											
**Citation [ref]**	**Random-Ization**	**Sample size Calculation and Required Number Included**	**Baseline Situation Similar**	**Blinding**	**Parameters of laser Use Described and Calculations Correct**	**Power-Meter Used**	**Numerical Results Available (Stats)**	**No Missing Out-Come Data**	**All Samples/Patients Completed the Follow-Up**	**Correct Inter-Pretation of Data**	**Total Score/10**
**LARGE SPOT SIZE**											
Aras [66]	Yes	Yes	Yes	Yes	No	No	Yes	Yes	Yes	Yes	8
Ustaoglu [67]	Yes	Yes	Yes	Yes	Yes	No	Yes	Yes	No	Yes	8
Asutay [68]	Yes	Yes	Yes	Yes	No	No	Yes	Yes	Yes	Yes	8
Ahrari [69]	Yes	Yes	Yes	Yes	Yes	Yes	Yes	Yes	Yes	Yes	10
Liang [70]	Yes	No	Yes	Yes	No	No	Yes	Yes	No	Yes	6
Amadori [71]	Yes	Yes	Yes	Yes	Yes	No	Yes	Yes	Yes	Yes	9
Caccianaga [72]	Yes	Yes	Yes	Yes	Yes	No	Yes	Yes	Yes	Yes	9
Gautam 2015 [73]	Yes	Yes	Yes	Yes	Yes	Yes	Yes	Yes	No	Yes	9
Gautam 2013 [74]	Yes	Yes	Yes	Yes	No	No	Yes	Yes	Yes	Yes	8
Nicotra [75]	Yes	Yes	Yes	Yes	No	No	Yes	Yes	No	Yes	7
Flieger [76]	Yes	Yes	Yes	Yes	Yes	No	Yes	Yes	Yes	Yes	8
Matys [77]	Yes	Yes	Yes	Yes	Yes	No	Yes	Yes	Yes	Yes	9
Feslihan [78]	Yes	Yes	Yes	Yes	No	No	Yes	Yes	Yes	Yes	8

**Table 2:** **Group A.** Small spot (0.02 cm^2^ to 0.08 cm^2^) analysis of papers. Outcome key (0–5): 0 = null effect; 1 = 10–20%; 2 = 20–40%; and 3 = 40–60%; 4 = 60–80%; 5 = 80%+. ΔOutcome Score represents the difference between the outcome scores of the laser treatment (T) and control (C) groups, with the latter subtracted from the former. **Key to abbreviations:** BMS = burning mouth syndrome, WH = wound healing, OM = oral mucositis, VAS = visual analogue scale.

Author (Ref)/Study Type	Number of Participants: Test (T) Control (C)	(i) Small Spot Size: Target Size cm^2^ (ii) Target: Superficial = 1 Deep = 2	Area Exposed to Tip (cm^2^); *Radiant Exposure* (Fluence J/cm^2^); Total Energy Delivered (Joules)	Net Outcome (ΔOutcome Score)	Dose Commentary	Statistical Analysis: Test *vs*. Control Group
Sugaya [41] RCT DB BMS	T = 13 C = 10	2.0 1	1.0 *0.24* N/R	0	Scanning technique 2 s/spot of 0.03 cm^2^ Dose insufficient	Test > control, although ns. VAS Remission of symptoms: test 46% control 40% N/S
Valenzuela [42] RCT B BMS	T gp.1 = 16. T gp.2 = 16. C = 12	2.0 1	0.3 *133/200* Gp. 1: 4.0/Gp 2: 60	1	Small area treated High radiant exposure, low volume exposed	Tests 1 and 2 > control: 15.7% vs. 7.3% VAS VAS score 16% improvement with test outcomes.
Barbosa [43] RCT DB BMS	T = 25 C = 19 C gp.2 (normal) = 8	2.0 1	N/R *3.0/4.28* N/R	0	Low irradiance (30 mW), multiple points/small spot size	Test = Control VAS Equivalence of laser to ALA
Dias [44] RCT DB WH	T = 16 C = 16	1.0 1	0.15 *3.0* N/R	3	Low irradiance (30 mW), multiple points to cover target	Test > control. D14 T = 16.4 (SD9.6) C = 26.2 (SD 10.6) D45 T = 5.9 (SD 1.9) C = 13.6 (SD 3.8) Wound Area D represents day 14 or 45. 40% reduction vs control T1 57% reduction vs. Control T2
Neves [45] RCT DB WH	T gp.1 = 18. T gp.2 = 18. C = 18	1.0 1	0.06 *3 /60* Gp 1: 0.9/Gp 2: 1.8	1	Low irradiance (30 mW), smaller area treated than target size	Test > control T 1 =14.4 (SD 5.1) T 2 = 14.3 (SD 6.1). C = 11.4 (SD 4) Measure: colourimetry, wound area. Significant: T1 (group 1 only)
Rezade [46] RCT B WH	T = 40 C = 42	6.0 2	0.28 *100* 28	0	Deep target, large area to treat: low dose to each point & small area treated	Test > Control N/S. T1 = 41.18 (SD13.03) C = 34.55 (SD 3.22) T2 = 36.6 (SD 7.98) C = 34.82 (SD 11.58) Measure: mouth opening T1 = Men T2 = Women
Tuk [47] RCT B Pain	T = 80 C = 83	1.0 2	0.08 *148.5* 11.88	0	Small area treated: dose to target insufficient?	Test = Control T = 4.1 (SD 2.4) C = 4.2 (SD 2.7) VAS
De Carli [48] RCT DB Pain	T (gp.1) = 11 T (gp.2) = 10 C = 11	6.0 2	0.28 *100* 28	0	Large and deeper target: small area treated. Dose low?	Test gp.1/2 = Control N/S Equivalence test gps to control VAS
Machado [49] RCT DB Pain	T = 42 C (gp.1) = 40 C (gp.2) = 20	6.0 2	0.2 *60* 12	1	Large and deeper target: small area treated. Dose low?	T= 1.6 C (gp.2 =1.1) *p* < 0.001 VAS
Magri [50] RCT DB Pain	T = 20 C (gp.1) = 21 C (gp.2) = 23	6.0 2	0.34 *5.9/7.5* Gp 1: 0.9/Gp 2: 1.4	0	Large and deeper target: small area treated. Dose low?	T = C (gp.1) T/C (gp.1) > C (gp.2) VAS C1 = Placebo C2 = No treatment
Ramalho [51] RCT B Pain	T (gp.1) = 15 T (gp.2) = 15 C (gp.1) = 15 C (gp.2) = 15	1.0 2	0.04 *4/40* Gp 1: 0.32/Gp 2: 3.2	0	Dose low	T = C (gp.1) T/C(gp.1) > C (gp.2) VAS C1 = Placebo C2 = No treatment
Oton-Leite [52] RCT DB OM	T = 15 C = 15	6.0 1	1.72 *6.2* 10.75	3	Oral mucositis High number of points low irradiance	T > C 50–75% reduction in OM in T vs. C OM Severity Test vs. Placebo
Ferrante [53] RCT B WH	T = 15 C = 15	6.0 2	0.08 *225* 18	1	Deep target, low dose	Measure: mouth opening Test: <swelling/trismus
Nobrega [54] RCT DB Pain	T = 30 C = 30	1.0 2	0.15 *38/76* Gp 1: 5/Gp 2: 2.3	3	Low dose but: high radiant exposure to apex 76 J/cm^2^	VAS Test *vs*. placebo no nil intervention
Marin Conde [55] RCT DB OM	T = 11 C = 15	6.0 1	2.6 *83.3* 216	3	Oral mucositis High radiant exposure large number of points	T > C 50% + reduction OM OM Severity Test vs. placebo no nil intervention
Silva [56] RCT DB OM	T =19 C =20	6.0 1	3.2 *4.0* 12.8	3	Oral mucositis Low radiant exposure large number of points	OM severity Test *vs*. Nil Marked reduction severity and incidence OM

**Table 3:** **Group B.** Medium spot (0.126 to 0.38 cm^2^) analysis of papers. Outcome key (0–5): 0 = null effect; 1 = 10–20%; 2 = 20–40%; and 3 = 40–60%; 4 = 60–80%; 5 = 80%+. **Key to abbreviations:** BMS = burning mouth syndrome, WH = wound healing, OM = oral mucositis, VAS = visual analogue scale. ΔOutcome Score represents the difference between the outcome scores of the laser treatment (T) and control (C) groups, with the latter subtracted from the former. SD, standard deviation.

Author (Ref)/Study Type	Number of Participants: Test (T) Control (C)	(i) Medium Spot Size: Target Size cm^2^ (ii) Medium Spot Size: Target Superficial = 1 Deep = 2	Area Exposed to Tip (cm^2^): *Radiant Exposure* (Fluence J/cm^2^) Total Energy Delivered (J)	Net Outcome (ΔOutcome Score)	Dose Commentary	Statistical Analysis: Test *vs*. Control Group
Arduino [57] RCT B OM	T = 18 C = 15	2.0 1	0.28 *10.7* 3.0	1	Incomplete data	Outset: VAS 3.35 (SD 2.18) Time (T4): 3.47. (SD 2.14)
Elbay [58] RCT DB Pain	T = 49 C = 49	1.0 2	0.6 *90* N/R	0	Target at depth, dose at surface too low?	Outset VAS 2.05 (SD 2.027). Post 0.11 (SD 0.727)
Ramirez [59] RCT DB Split Mouth Pain	T = 20 C = 20	6.0 2	3.14 *1.55* 12.8	0	Scanning technique to cover area, target at depth: dose too low?	VAS Outset 52.47 (SD 7.05) T (24 hours) 30.74 (SD 8.94)
Landucci [60] RCT DB Split Mouth Pain	T = 22 C = 22.	6.0 2	0.126 *2.39* 0.3	1	Target at depth, large target: dose low	VAS outset 0.27. T2 = 3.86
Moosavi [61] RCT DB Pain	T (gp.1) = 14 T (gp.2) = 12. C = 15	1.0 2	0.25 *12.0* 3.0	3	Best effect with 810 nm. Small target (pulp), and good transmission via (dentine)	VAS outset 21.11 (SD 18.19). Time 1: 51.94 (SD 20.8) Time 2: 17.77 (SD 13.57).
Amanat [62] RCT DB Pain	T = 30 C = 30	6.0 2	0.283 *12.73* 3.6	0	Large target at depth: dose too low	VAS outset: 7.5 (SD 2.3). VAS post Rx: 3 (SD 3.7)
Shirani [63] RCT DB Pain	T = 8. C = 8	6.0 2	0.6 *6.2* 6.3	1	Multiple points (number not specified)	VAS outset 4 (SD 1.5). VAS post Rx 2.5. (SD 1.5)
Ang Khaw [64] RCT DB Split Mouth WH	T = 20 C = 20	1.0 2	0.26 *3.6* 8.0	0	Too low: 7.6 J total to sub-surface target	Wound area VAS outset 0.27 T2: 3.86. T3: 1.41
Antunes [65] RCT DB OM	T = 47 C = 47	6.0 1	17.28 *4.2* 72.0	4	Oral mucositis: multiple points large area	OM severity Incidence OM Grade 3 C = 40.5% grade 1/2 = 21.3%

**Table 4:** **Group C.** Large spot (0.51 cm^2^–4 cm^2^) analysis of papers. Outcome key (0–5): Outcome key (0–5): 0 = null effect; 1 = 10–20%; 2 = 20–40%; and 3 = 40–60%; 4 = 60–80%; 5 = 80%+. **Key to abbreviations:** BMS = burning mouth syndrome, WH = wound healing, OM = oral mucositis, OTM = orthodontic tooth movement, VAS = visual analogue scale, EPT = electric pulp test, IS = implant stability.D = day number at intervention. SD, standard deviation. ΔOutcome Score represents the difference between the outcome scores of the laser treatment (T) and control (C) groups, with the latter subtracted from the former.

Author (Ref)/Study Type	Number of Participants: Test (T) Control (C)	(i) Large Spot Size: Target Size cm^2^ (ii) Large Spot Size: target Superficial = 1 Deep = 2	Area Exposed to Tip (cm^2^): *Radiant Exposure* (Fluence J/cm^2^) Total Energy Delivered (J)	Outcome (ΔOutcome Score)	Dose Commentary	Statistics Control Group
Aras [66] RCT B WH	T = 32 C = 16	2.0 2	3.0 *4.0* 12	2	Incomplete data 3rd molar extractions measures: inter- incisal opening	Measure: mouth opening C-D0: 45. C-D2: 21.1 (SD5.2) C-D7: 29(SD 6.2)
Ustaoglu [67] RCT DB WH	T = 20 C = 20	1.0 1	2.8 *2.86* 8.0	1	Gated mode peak power 3 x average Gaussian beam	Wound area C -D0: 0 C-D14: 82. C-D21: 0 (H2O2)
Asutay [68] RCT DB WH/Pain	T = 15 C (gp.1) = 15 C (gp.2) = 15	2.0 2	3.0 *4.0* 12	1	Large target at depth: dose low. Control gp.3 placebo	Measure: mouth opening. VAS T > C for VAS reduction
Ahrari [69] RCT DB WH/Pain	T = 10 C = 10	6.0 2	1.76 *3.4* 6.0	1	Large target at depth: dose low. Placebo vs. Test VAS / inter-incisal	Measure: mouth opening. VAS C -D0: 26.9 (SD 7.78) C-D56: 29.36 (SD 6.46)
Liang [70] RCT DB Pain	T = 30. C = 30	1.0 2	1.0 *3.6* 3.6	3	Small sub-surface target (pulp), good optical transmission (dentine)	EPT C = 1.9%. (T = 52.8%)
Amadori [71] RCT DB OM/Pain	T = 62 C = 61	6.0 1	1.0 *4.5* 4.5	1	Treatment target analgesia: dose low	OM severity. VAS C >T
Caccianaga [72] RCT DB OTM	T = 18 C = 18	6.0 2	6.0 *24* 150	3	Sub-surface target: dose optimal (flat top beam profile)	OTM Alignment D: C = 284.1. Test D: 211.8
Gautam 2015 [73] RCT DB Om/Pain	T = 23 C = 26	6.0 1	12 *3.0* 36	3	Oral mucositis: large target at surface	OM severity OM C >> T VAS C > T
Gautam 2012 [74] RCT TB OM/Pain	T =115 C = 124	6.0 1	6.0 *3.5* 21	4	Oral mucositis: large target at surface	OM severity. VAS OM C = 77/110 ( T= 25/110) (Pain C >>T
Nicotra [75] RCT B Pain	T = 19 C = 37	1.0 2	1.0 *30* 30	2	Ortho pain 3 × 10 s Test/Control C1 and C2(placebo)	VAS C > T
Flieger [76] RCT B Implants	T = 20 C = 20	1.0 2	0.5024 *40* 20	3	Implant stability	IS C << T
Matys [77] RCT Implants	T = 12 C =12	1.0 2	0.5024 *40* 8.0	1	Implant stability	IS C < T
Feslihan [78] RCT B WH/Pain	T= 30 C =30	2.0 2	3.0 *6.0* 18	0	Third molars test vs prednisolone. No control: measure of steroid vs. Laser: equivalence	Measure: mouth opening. VAS C = T
